# Genito-Urinary and Syphilitic Diseases

**Published:** 1902-05

**Authors:** Byron H. Daggett

**Affiliations:** Buffalo, N. Y.


### PROGRESS IN MEDICAL SCIENCE.

### Genito-Urinary and Syphilitic Diseases.

Conducted by BYRON H. DAGGETT, M.D., Buffalo, N.Y.

### THE UNRECOGNISED CHANCRE.

W. S. Gotthiel (*International Medical Magazine*, October, 1901,) calls attention to the frequent insignificance and fugacity of the initial syphilitic lesion which often leads to its nonrecognition.

Points of diagnosis: (1) The original lesion at its beginning is a round cell accumulation in the skin or submucous tissue. Ulceration usually follows: appreciable induration invariably results; (2) the tumor is indolent, painful and refractory to treatment; (3) there is a characteristic "stony" induration of the nearest lymph glands, which differs from the general adenopathy of the systemic infection; (4) chancre runs its course in a few weeks, while tuberculosis takes months and carcinoma even years for development; the signs of this disease, ostectopic pain, cephalalgia, synovitis, general lymphadenitis, exanthema, and the like, must be carefully searched for. They may be overlooked in a careless examination.

---

### THE CURABILITY OF SYPHILIS.

Gotthiel, William S. (*International Medical Magazine*, October, 1901,) states that everyday experience shows that the great majority of cases are cured. He concludes: (1) syphilis is a curable disease and under restrictions a self-limited one; (2) the chances of a cure, after a certain time, are so great it may be claimed that it has been effected; (3) a patient who has been properly treated throughout the active stages of this disease, and who presents no manifestations of its persistence after the lapse of several years may be pronounced cured.

